# China’s Accession to the WTO as a Shock to Residents’ Health—A Difference-in-Difference Approach

**DOI:** 10.3390/ijerph192214728

**Published:** 2022-11-09

**Authors:** Yiping Sun, Chengjun Wu, Xiaoming Zhu, Pingguan Bian

**Affiliations:** 1 Hubei Enterprise Culture Research Center, Hubei University of Economics, Wuhan 430205, China; 2 School of Business Administration, Zhongnan University of Economics and Law, Wuhan 430073, China; 3 School of Economics and Trade, Guangxi University of Finance and Economics, Nanning 530003, China; 4 Hubei Yihua Group, Institute of International Business, Zhongnan University of Economics and Law, Yichang 443099, China

**Keywords:** trade shock, residents’ health, WTO, China

## Abstract

The impact of regional trade shocks on population health has been a topic of interest in health economics in recent years. Unfortunately, there are few studies directly discussing the impact of regional trade shocks caused by China’s WTO accession on the health of Chinese residents, which is essential to explore the connection between a country´s opening to international trade and the health of its residents. Taking China’s accession to the WTO as a quasi-natural experiment, based on the micro individual samples of the China Health and Nutrition Survey (CHNS) from 1993 to 2011, this paper, for the first time, studies the impact of regional tariff uncertainty caused by China’s accession to the WTO on the health of Chinese residents and its mechanisms by adopting the Difference-in-Difference (DID) model. The study finds that compared with the areas initially facing low tariff uncertainty, the areas with high initial tariff uncertainty have a greater negative impact on the health of residents after China acceded to WTO, which means that the trade shock caused damage to the health of residents. After a series of tests on the effectiveness and robustness of DID, this conclusion is still valid. The impact of the trade shock on residents’ health varies with the type of residence, gender, and geographical location, and there is a nonlinear relationship. Further mechanism tests show that the trade shock has worsened the health status of residents through rural migration channels, working hours channels, and pollution emission channels. This study provides micro evidence for objectively evaluating the health effects of trade shock and has important implication for considering the health loss of Chinese residents in the process of trade liberalization.

## 1. Introduction

Health is the source of people’s well-being, the most precious human capital, and an important symbol of national prosperity and national wealth. Economic development is one of the important factors affecting the health of a population [1]. Since its reform and opening-up, China has seized the historical opportunity to participate in economic globalization, providing it with a solid foundation for the development of its economy and for a better quality of life of its people. One indicator of better nutrition (and likely improved quality of life) is that the average height of 19-year-old males in China jumped to first place in East Asia in 2019 [2]. As the problem of physical development and under-nutrition of residents has been continuously improved, the Chinese government has attached great importance to the development of national health and made strategic plans to comprehensively promote residents’ health. However, some official statistical results show that the health status of Chinese residents is still a critical issue that cannot be ignored.

At the same time, much literature has shown that trade liberalization is an important economic factor affecting the health of the population [3,4,5]. Among this research, the permanent normal trade relations treatment granted by the U.S. after China’s accession to the WTO (this historical event is expressed in the literature as a “reduction in trade policy uncertainty”; since this paper finds that this event was detrimental to the health of the Chinese residents, we use the term “trade shock” throughout the text) is one of the most important factors of trade liberalization. Given the seriousness of the health problems of Chinese residents and the possibility of economic factors playing an important role in this, in the context of COVID-19 ravaging the world, the rise of trade protectionism, the increasingly complex international environment, and the obvious increase in instability and uncertainty, re-examining the health effects of trade liberalization from the perspective of tariff uncertainty is important for clarifying the hidden costs in trade opening and promoting the development of China’s health.

The impact of regional trade shocks on health has been one of the topics of interest in health economics in recent years. Unfortunately, we find few studies directly related to the impact of regional trade shocks caused by tariff uncertainty on the health of Chinese residents. Using data from the China Health and Nutrition Survey (CHNS) from 1993–2011, this paper systematically examines the impact of permanent normal trade relations (PNTR) tariff treatment provided by the United States after China’s formal accession to the WTO to answer the following questions: (1) What is the impact of regional trade shock on the health of Chinese residents? What is the extent of the impact on regions facing different tariff uncertainty changes? (2) Does the impact of regional trade shock on the health of the residents vary by type of residence, gender, and geographic location? Is there a non-linear characteristic of this effect? (3) What are the mechanisms of the effect of regional trade shock on the health of the residents?

The remainder of this paper is organized as follows: Section 2 is a literature review; Section 3 presents the background and theoretical hypotheses based on existing relevant studies; Section 4 presents the model setting and variable description; Section 5 and Section 6 includes an empirical analysis, robustness test, and heterogeneity analysis of trade shock on the health status of Chinese residents; Section 7 includes a mechanism test of trade shock on the health status of Chinese residents; Section 8 is the conclusions.

## 2. Literature Review

### 2.1. Studies on the Economic Effects of Trade Policy Uncertainty

Since the background and measurement of trade shock in this paper are the same as trade policy uncertainty in the existing literature, it is necessary to review the literature on trade policy uncertainty. Since Handley [6] and Handley and Limao [7] pioneered the field of trade policy uncertainty, many scholars have conducted comprehensive, detailed as well as systematic research work on its possible economic effects. First, for the trade activity itself, which is most closely linked to TPU, the literature has more often affirmed the positive role of declining TPU in promoting a country’s export, and further studies have shown that this expansion benefits from the joint progress of the intensive and extensive margin [7,8], but the dominated margin of export growth is still not clear. For example, in the context of China’s WTO accession leading to the granting of PNTR by the United States, China’s export expansion to the United States was mainly driven by the extensive margin [9]. If this decline in trade policy uncertainty is caused by China’s accession to the Regional Comprehensive Economic Partnership (RCEP) and the Trans-Pacific Partnership Agreement (TPP), then the intensive margin will dominate [10]. At the same time, the impact of decreasing TPU on the trade sector is also reflected in many aspects such as decreasing prices of export products [9], improving the quality of import products [11] and domestic value added [12], which also has a positive impact on China’s import expansion that should not be ignored [11]. Second, for the macro effects of TPU, Pierce and Schott [13] used business panel data from the U.S. Census Bureau from 1990–2007 to find that the PNTR preferences provided by the U.S. to China severely hurt U.S. manufacturing jobs, and the greater the TPU faced by the industries, the greater the employment losses suffered after the tariff cuts. In contrast, declining trade policy uncertainty boosted the number of employees in Chinese firms by expanding the number of firms [14,15]. Further, the decline in trade policy uncertainty through trade agreements has an important contribution to the world economic recovery [16]. Third, for the micro impact of trade policy uncertainty, the literature focuses on the individual firm perspective, and the scope of research mainly includes firm productivity [17,18], firm product innovation [19], firm purchasing patterns [20], and firm investment [21,22]. Through the three strands of the literature, it is easy to find that the existing literature on the micro effects of TPU is mostly limited to the firm dimension, but there are relatively few studies examining its effects from the individual point of view. On the one hand, this is because there is no complete database at the microeconomic level, and the available individual data are mostly limited to sample surveys in individual regions, which makes the generalizability of the findings based on such data controversial; on the other hand, the existing mainstream measures of TPU rely on industry-specific tariff differentials, which are difficult to match with individual survey data. Some literature shows that the central-eastern region of China is the main region affected by differentials. Therefore, for the former issue, this paper overcomes it by using CHNS survey data, which has a major sample of respondents from central-eastern China, and it is of general interest to identify the causal effects of trade shock through this micro survey data. For the latter issue, the Shift-Share method proposed by Bartik [23] provides a feasible condition for this paper to construct a regional dimension of trade shock.

### 2.2. Studies on the Health Effects of Trade Liberalization

Most closely linked to this paper are studies related to the health effects of trade liberalization. From an aggregate perspective of trade across the world, Owen and Wu [24] give the first empirical evidence on the impact of trade on health at the aggregate level, based on a panel of 219 countries at 5-year intervals from 1960–1995, showing that trade growth contributes to health development along two dimensions: increased human life expectancy and reduced infant mortality. However, these effects are not significant in developed countries. Similarly, Stevens, Urbach and Wills [5] found that the health benefits of trade liberalization decreased as a country’s income level rose and even showed negative effects in high-income countries. Further, Herzer [25] reassessed the long-term health effects of trade using trade data for 74 countries over a period of up to 50 years, and the results similarly demonstrated that the positive effects of trade liberalization on life expectancy were concentrated in less developed countries. In recent years, a large portion of the literature has provided empirical evidence on the effects of trade liberalization on population health and mortality in the United States, Mexico, Myanmar, and Brazil based on the perspective of import liberalization due to tariff reductions [26,27,28,29,30,31], but the current empirical studies based on China have yet to be expanded. Based on a quasi-natural experiment of China’s WTO accession, Fan, et al. [32] found that input tariff cuts adversely affected workers’ health by increasing working hours and also further widened the earnings and health gap between skilled and unskilled workers.

Undeniably, in-depth studies have been conducted on the effects of trade liberalization and health from multiple perspectives, which provide useful research ideas and paradigms for this paper. However, there is a gap in the literature regarding the impact of regional trade shocks on the health of the Chinese population. Given this, this paper may have the following contributions: First, we broaden the research perspective. While the literature has focused on the health effects of import shocks in a country, this paper is a first step in identifying the economic effects of regional tariff uncertainty on the health of Chinese residents from the perspective of export shocks caused by China’s WTO accession, which is a useful attempt in this area. Second, based on the heterogeneous impact of the trade shock on different cities, we attempt to construct regional-level trade shock indicators to explore its regional heterogeneous effects, which helps to understand the dynamic linkage between the regional trade shock and residents’ health and the inherent transmission mechanism; third, we adopt cutting-edge research methods. Compared with the least squares method that has been commonly used in the literature to explore the average treatment effect, we adopt the Difference-in-Difference (DID) method, complemented by the continuous treatment group identification of regional trade shock, to identify the average and heterogeneous effects of trade shock more precisely on the health of Chinese residents.

## 3. Background and Theoretical Hypotheses

### 3.1. Background

Since 1986, China has been trying to return to the General Agreement on Tariffs and Trade (GATT) and the subsequent World Trade Organization (WTO); a fundamental principle of GATT/WTO is to grant Most Favored Nation (MFN) treatment among Members. Although the U.S. granted MFN to China as early as 1979, it attached a series of requirements that the U.S. would have to review and reaffirm annually. Once China does not receive MFN treatment in a given year, the U.S. will impose higher non-MFN tariffs on China. Thus, the U.S. initially granted China temporary MFN treatment, and during its WTO accession negotiations, China requested that the U.S. eliminate the annual review of the granting of normal trade relations status to China. In March 2000, Clinton asked the U.S. Congress to commit to permanent normal trade relations (PNTR) status for China. In May 2000, the U.S. agreed to grant China PNTR status. Although legislation supporting China’s PNTR status was enacted, it became effective only after China’s accession to the WTO. In sum, the threat of higher tariffs on its exports to the United States was removed after China’s accession to the WTO, and tariff uncertainty for that product declined, providing a quasi-natural experiment for the policy assessment in this paper.

### 3.2. Migration

Trade liberalization is likely to facilitate the movement of residents from less developed areas to more developed areas, thereby affecting the population’s health status. The need for export expansion following China’s WTO accession creates more local employment opportunities, thus attracting less-developed rural populations to cities, especially to cities with higher trade openness [33]. However, rural migrants face serious inequalities of opportunity, such as difficulties in obtaining a local household registration (hukou), acquiring a stable residence, and accessing lower-cost healthcare coverage [34]. In particular, during the sample period of this paper, migrant workers might prefer to pay for medical insurance and seek medical treatment in their original hukou location because the system of medical insurance processing and settlement in different places has not been built, and the high medical expenses might hinder migrant workers’ access to better medical services in cities. This widespread inequality of opportunity seriously increases the psychological stress of migrant workers, which in the long run is detrimental to the development of the health status of migrant workers in industries directly affected by trade policy shocks, such as manufacturing. Based on the above discussion, trade shock caused by China’s WTO accession is likely to affect the health status of migrant workers, thus causing overall negative health effects of trade.

**Hypothesis** **1.** *China’s WTO accession leads to an outflow of the rural labor force and damages the health status of residents who have not obtained urban hukou through inequality of opportunity caused by the hukou system.*

### 3.3. Working Hours

A decline in tariff uncertainty due to China’s WTO accession leads not only to the entry of exports by new firms but also to an increase in the size of exports by incumbent firms [7,8]. In order to expand exports, firms need to hire more labor on the one hand and increase the working hours of existing workers on the other. Ouyang and Yuan [15] show micro-empirical evidence based on data from Chinese industrial firms that, following a decline in trade policy uncertainty, firms increased the number of their employees to meet an increasingly dramatic expansion of their export product range. Stable employment on the one hand raises the income level of the population [35] and reduces the probability of health illnesses due to anxiety about economic risks [36,37]. However, the employment gains resulting from export expansion have been more concentrated in the manufacturing sector, where China has a comparative advantage, and the related problem of excess labor hours, which is common in processing and manufacturing firms, also poses a serious threat to the development of health status of the population. For example, Burgoon and Raess [38], based on survey data from European multinationals, found that trade volume growth increased the working hours of employees in import-competing sectors, to the detriment of their physical health. More directly, Facchini, et al. [33] proved that the reduction in trade policy uncertainty led to longer working hours of unskilled workers. The increase in working hours reduces the necessary time for physical exercise and sleep breaks, which has a significant negative impact on both the physical and mental health of the population [39].

**Hypothesis** **2.** *China’s WTO accession increases the average number of hours worked by residents while providing more employment opportunities, thereby crowding out necessary exercise and sleep and further contributing to the deterioration of residents’ health.*

### 3.4. Environmental Pollution

In addition to the above-mentioned channels, environmental pollution is also a key mechanism where the trade shock affects residents’ health. Clearly, the decline in tariff uncertainty caused by China’s accession to the WTO is an important form of trade liberalization. The previous literature suggests that trade liberalization acts on the environmental quality of host countries through two main channels: first, according to the analysis of “the Porter Hypothesis”, trade liberalization reduces the cost of introducing technologies related to environmental protection and lowers the threshold for host country firms to absorb green technologies and carry out green innovation activities, which to some extent improves the local environmental governance capacity [40]. Second, according to the “Pollution Paradise Hypothesis”, developing countries, in the process of promoting trade liberalization, often enact lax environmental regulations at the initial stage to attract the transfer of foreign industrial chains as much as possible, thus reducing the environmental costs required for foreign enterprises to invest and produce in the host country [41]. Specifically, after China acceded to WTO, it has taken over many low-end manufacturing industries from Europe and the United States, or other places, and has gradually become a global processing and production center for consumer goods, with distinct industrial characteristics of serving external demand with internal production. Third, as Handley [6], Handley and Limao [8] and Feng et al. [9] point out, declining trade policy uncertainty leads to lower barriers to export entry, more low-productivity firms start to export, and production expansion of inefficient firms will cause more environmental pollution and poorer working and living conditions. An empirical study based on 274 prefecture-level cities in the relevant literature showed that export trade expansion increased the emission concentrations of pollutants such as sulfur dioxide, nitrogen oxides, and respirable particulate matter in China, resulting in the deterioration of regional environmental conditions and increasing the risk of disease and mortality among residents [4].

**Hypothesis** **3.** *China’s WTO accession will prompt the transfer of low-end manufacturing industries from abroad and lead to more exporting entrants of low productivity, which will increase the concentration of local pollutant emissions and be detrimental to the development of the health status of residents.*

## 4. Model and Variables

### 4.1. Empirical Model

To precisely identify the policy effects of the U.S. government’s granting of PNTR treatment on the health of Chinese residents after China accedes to the WTO, and in view of previous empirical findings, we treat China’s accession to the WTO as a quasi-natural experiment and use the DID method to construct the following basic econometric model:

(1) $$health_{icy}=\alpha +\beta TScity_{c}\times Post02_{y}+{X^{\prime }}_{icy}^{}\gamma +\delta_{c}+\delta_{y}+\varepsilon_{icy}$$

where i represents the individual, c represents the city, and y represents the year observation; the dependent variable $health_{icy}$ is the health status of individual i in year y, which is measured by a binary indicator of whether individual i has hypertension, and is supplemented by other health indicators for robustness testing; $TScity_{c}$ is the degree of trade shock using the tariff differential at the prefecture level in 2001, whose detailed construction will be explained in the next section; $Post02_{y}$ is a dummy variable for the year of China’s accession to the WTO, and it is recorded as 1 if y⩾2002, otherwise it is 0; the interaction term $TScity_{c}\times Post02_{y}$ is the core explanatory variable of this paper, and its coefficient β portrays the average difference between the health status of residents in high and low tariff differential prefectures before and after China’s accession to WTO, which is also the causal effect of the trade shock on the health of residents; $\delta_{c}$ and $\delta_{y}$ are prefecture and year fixed effects, respectively, to control for time-invariant prefecture differences and the effect of common economic cycles faced by different prefectures on the health status of residents. In order to obtain unbiased estimates of the coefficients, a vector of individual-level characteristic variables should be included in the model in addition to city and year fixed effects. α, β, γ are parameters to be estimated. To avoid serial correlation and heteroskedasticity, we cluster the standard errors at the province level [42].

### 4.2. Variables

1. Status of residents’ health ($health_{icy}$). The advantage of objective indicators over subjective indicators is that there is a uniform assessment standard across individuals. Since the beginning of the survey in 1991, CHNS has conducted three rounds of blood pressure measurement for each respondent, which provides data support for the objective evaluation of the individual health status of respondents. To avoid measurement bias resulting from the initial results being influenced by psychological fluctuations and other factors, we use only the mean values of the last two blood pressure measurements to determine whether an individual has hypertension. Respondents with systolic blood pressure greater than 120 mmHg or diastolic blood pressure greater than 80 mmHg were considered hypertensive according to the criteria recommended in the guidelines for the treatment of hypertension in China. If an individual had hypertension, it was recorded as 1, and vice versa as 0. (The regression results remain robust when we use standard errors an individual is away from the mean value of the prefecture as the dependent variable).
2. Trade shock ($TScity_{c}$). Trade shock can be expressed as the magnitude of an adverse change in trade policy when that change occurs. Consistent with existing studies, we measure this change using the difference between the US two-column tariff and the MFN tariff rate before China acceded to the WTO [13]. Specifically, we calculate the tariff differential based on the US’s HS-8-digit code ad valorem rates, averaged to the HS-6-digit. Next, the tariff differential at the industry level for each 4-digit of the National Economic Classification can be obtained based on the HS-CIC code conversion table provided by Brandt et al. [43]. Finally, if tariffs fall more in industries with a higher share of employment in a region, the greater the decline in tariff uncertainty in that region, and thus the initial employment structure of each region is an important determinant of its exposure to shocks. Referring to the approach of Bartik [23], we sum the industry-level tariff differentials to the prefecture level according to the following equation:
  (2) $$TScity_{c}=\sum_{j}\frac{employment_{cj}}{employment_{c}}TS_{j}$$
  where $employment_{c}$ is the total employment in the region c in 2001; $employment_{cj}$ is the employment in the industry j in the same year. We identify the treatment group through the continuous variable $TScity_{c}$ because: (1) the greater the tariff uncertainty faced by a prefecture, the greater the impact of China’s WTO accession to permanent normal trade relations on the health status of the region’s residents; (2) the U.S. 2-column tariff was set in 1930, and the tariff differential before the implementation of the policy is highly exogenous. More specifically, China’s accession to the WTO requires the consent of all Members, and many details of the negotiated agreement remained unresolved until 2001, and the timing of WTO accession is unpredictable, i.e., China’s accession to the WTO is an exogenous event. The literature evaluating the trade and economic effects of trade policy uncertainty points out that the Smoot–Hawley tariffs imposed by the US have long been determined by history, suggesting that the tariff uncertainty is exogenous [9,13,19]. Therefore, the endogeneity problem can be avoided to a large extent. (3) The prefecture employment structure data used in this paper were established before China’s WTO accession, and the concomitant expansion of exports is a channel of influence linking trade liberalization to individual health, which will be explored in more detail below.
3. Year dummy variable of WTO ($Post02_{y}$). China formally joined the WTO on December 11, 2001, and its import tariffs on goods were significantly reduced, while it received the corresponding preferential import tariff treatment from the U.S. PNTR, and the tariff uncertainty it faced declined. In this paper, the year 2002 is used as the actual effective year of the policy, and the value of 1 is assigned if the sample is in the year 2002 or later, otherwise it is 0.
4. Individual-level characteristic variables (${X^{\prime }}_{icy}^{}$). Residents’ health will inevitably be influenced by individual-level factors; concerning the relevant literature, the following control variables are introduced: (1) Gender (gender). The value is 1 when the respondent is male, otherwise, it is 0. (2) Age (lnage). It is expressed as the logarithm of the difference between the year the respondent was interviewed and his or her calendar year of birth. (In semi-logarithmic form, the effect of age on health is $\frac{\partial h^{-}}{\partial \ln a}=\frac{\partial h^{-}}{\partial a}\frac{\partial a}{\partial \ln a}=\frac{\partial h^{-}}{\partial a}\cdot a$. The results are unaffected when we do not take the logarithm of the age). (3) Educational background (eduback): The CHNS database records the highest educational level obtained by each respondent, and the questionnaire results include seven types of results: never educated, graduated from elementary school, graduated from junior high school, graduated from high school, graduated from secondary school, graduated from college or university, and graduated from master’s degree or above. We assign values 0~6 to eduback in order. (The results remain robust when we replace the categorical variable with a series of dummy variables and their interaction terms with $Post02_{y}$). (4) Whether the respondent has medical insurance (insurance). The variable takes the value of 1 when the respondent has health insurance and 0 otherwise. (5) Marital status (marry). CHNS gives the marital status of each respondent in that year, and distinguishes five types of states: unmarried, married, divorced, widowed, and separated, and assigns values from 1 to 5 in that order. (Similar to the variable of educational background, the results remain robust when we replace the categorical variable with a series of dummy variables and their interaction terms with $Post02_{y}$).

### 4.3. Data

The first data set used in this paper is from the China Health and Nutrition Survey (CHNS) collected by the Institute of Nutrition and Food Safety of the Chinese Center for Disease Control and Prevention (CDC) in collaboration with the University of North Carolina, USA. These data cover a large number of micro-individuals. However, due to serious data deficiencies in the early years, only seven rounds of data from 1993–2011 were used, covering 41 cities in Shanghai, Beijing, Shandong, Guangxi Zhuang Autonomous Region, Jiangsu, Henan, Hubei, Hunan, Guizhou, Liaoning, Chongqing, and Heilongjiang Provinces. Excluding the sample with missing core variables yields unbalanced panel data for 1993–2011, containing 11,332 surveyed residents and 55,320 valid individual observations. (Because the decline in tariff uncertainty due to China’s WTO accession occurred in 2002, the sample from 1993 to 2011 is reasonable for our research questions. Similarly, based on China’s WTO accession, Pierce and Schott (2016) [13] use the sample from 1990 to 2007 to investigate the impact of the decline in trade policy uncertainty on employment; Liu and Ma (2020) [19] use the sample from 1995 to 2007 to investigate the impact of the decline in trade policy uncertainty on innovation). The second data set comes from the 2001 China Industrial Enterprises Database provided by the National Bureau of Statistics of China and the 2001 China Customs Database provided by the General Administration of Customs for the employment and export weights used in the construction of the prefecture-level tariff uncertainty indicators. The third data set is from the China Regional Statistical Yearbook for all years, which is used to construct prefecture-level characteristic variables. The rest of the data are obtained from the CSMAR and the EPS database. The descriptive statistics of the main variables are shown in Table 1.

## 5. Empirical Results

### 5.1. Baseline Specification

The results of the baseline specification of trade shock on the health status of the population are reported in Table 2. Column (1) contains only prefecture and year fixed effects, and the results show that the coefficient on the interaction term $TScity_{c}\times Post02_{y}$ is significantly positive, which indicates higher excessive blood pressure and hence worse health. Column (2) further introduces a full set of individual-level control variables, and the estimated results are still positive, but the coefficient is slightly larger compared to column (1), reflecting that the omitted variables at the individual level are likely to cause the treatment effect to be underestimated. The above results show that prefectures that had faced higher tariff differentials after China’s accession to the WTO had higher rates of morbidity and a higher degree of negative impact on the health status of the population compared to prefectures with smaller ones, so it can be argued that China’s WTO accession weakened the health status of the residents. Considering the literature showing that the difference in the level of public service provision across regions may have a significant impact on the health of residents, this paper selects fixed asset investment (lnfinv00×Post02) in each prefecture to measure the level of public services and introduces the interaction term between the level of regional public services before China’s WTO accession in 2000 and the policy dummy. The interaction term is introduced into the model (1) to mitigate the possible trends of different residents’ health status due to regional characteristics, as shown in column (3), where the significance levels of the core explanatory variables do not change. The coefficients on the interaction term $TScity_{c}\times Post02_{y}$ in columns (1)–(3) have highly significant and close statistical and economic significance. In column (3), for example, the estimated coefficient is 0.00354, indicating that for every 1% increase in regional initial tariff differential, the average rate of residents will increase by about 2.6% after China’s accession to the WTO. One possible explanation is that after China’s accession to the WTO, regions that had faced higher tariff differentials received higher scale tariff reductions, and industries closely related to exports expanded rapidly, but the slow improvement in factory working conditions and the increase in working hours, combined with the overlap of work content dominated by repetitive mechanical labor, created a greater impediment to the development of residents’ health status. Finally, based on the estimation results of the control variables, the author found that men and older respondents have worse health status, which is consistent with objective facts and economic intuition. In addition, the higher the educational background of the residents, the better their health status, which is also consistent with the findings of existing studies [44]. Interestingly, the coefficient of insurance is significantly positive, indicating that those who have insurance are in worse health, a result consistent with the theory of adverse selection and moral hazard in microeconomics.

### 5.2. DID Validity Test

The validity of the DID method rests on several important assumptions; firstly, the common trend assumption for the treatment and control groups needs to be satisfied, and secondly, it needs to be tested whether the policy has a significant expected effect or is influenced by other unobservable factors.

#### 5.2.1. Dynamic Effect

**Figure 1:**
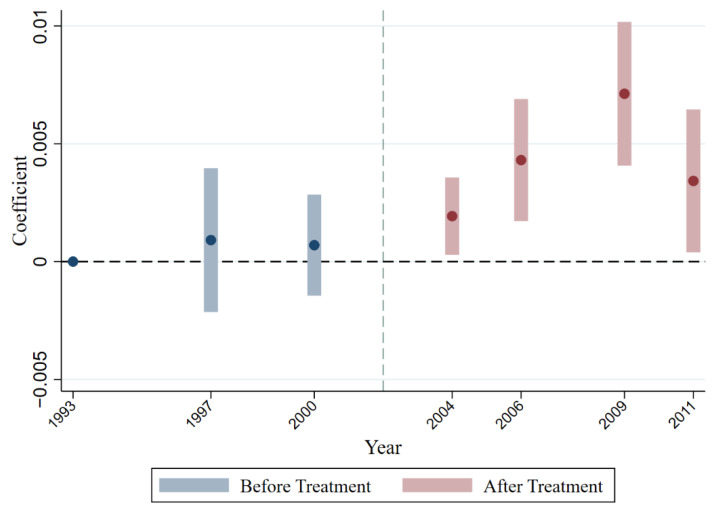
Illustration of the common trend prior to accession to the WTO.

The common trend assumption is one of the important conditions to be satisfied by the DID method, which means that the health status of the residents in the treatment and control groups should change according to the same trend before they have been subjected to a policy shock, while a significant difference will arise after the policy takes effect [45]. The specific test model is as follows:

(3) $$health_{icy}=\alpha +\sum_{k=1993}^{2011}\beta_{k}TScity_{c}\times Year_{k}+X_{icy}^{\prime }\gamma +\delta_{c}+\delta_{y}+\varepsilon_{icy}$$

where $Year_{k}$ represents the time dummy variable for each year of the sample period, and $Year_{k}$ takes 1 if it is the k year, otherwise, it takes 0. The first year of the sample is excluded as the base year in the regression to exclude the full multicollinearity problem; the rest of the symbols are the same as in model (1). This section is most concerned with the estimated coefficient of the interaction term $TScity_{c}\times Year_{k}$. The magnitude and significance of the estimated coefficient β before the year of the policy shock can determine whether there is a significant difference between the treatment and control groups, and whether the parallel trend hypothesis is satisfied. As shown in column (1) of Table 3, testing these interaction terms reveals that the policy effects of trade shock on the health status of the population emerge only in 2004, 2006, 2009, and 2011 after China’s accession to the WTO, while the coefficients pertaining to the years before accession do not significantly differ from zero. This supports the common trend hypothesis (see also Figure 1).

#### 5.2.2. Expectation Effect

The DID requires that there is no expected effect before the actual policy shock occurs to ensure the exogeneity of this policy identification, thus avoiding the assessment of the individual’s expected effect on the actual implementation of the policy in this paper before the policy is implemented. In this regard, we add $TScity_{c}\times Pre1_{y}$ in model (1), which is an interaction term between the dummy variable and the treatment group variable in the year before the policy. (The results remain robust when we include interaction terms for the four-year (i.e., 1997 to 2000) dummies with the variable TS, indicating that there are no expected effects). If the estimated coefficient of this interaction term is not significant, it indicates that there is no potential expected effect before the policy shock. As shown in column (3) of Table 3, the coefficient of this term is insignificant and close to zero, indicating that the residents in this paper did not form expectations of health status adjustment before China’s WTO accession. There were no significant expected benefits. At the same time, the coefficients of the core explanatory variables of interest $TScity_{c}\times Post02_{y}$ in this paper do not change substantially compared to the results of the benchmark regression when considering the possible existence of the expected effect. This result suggests that the policy shocks resulting from China’s implementation of WTO accession are strongly exogenous to the health status of regional residents.

#### 5.2.3. Placebo Test

**Figure 2:**
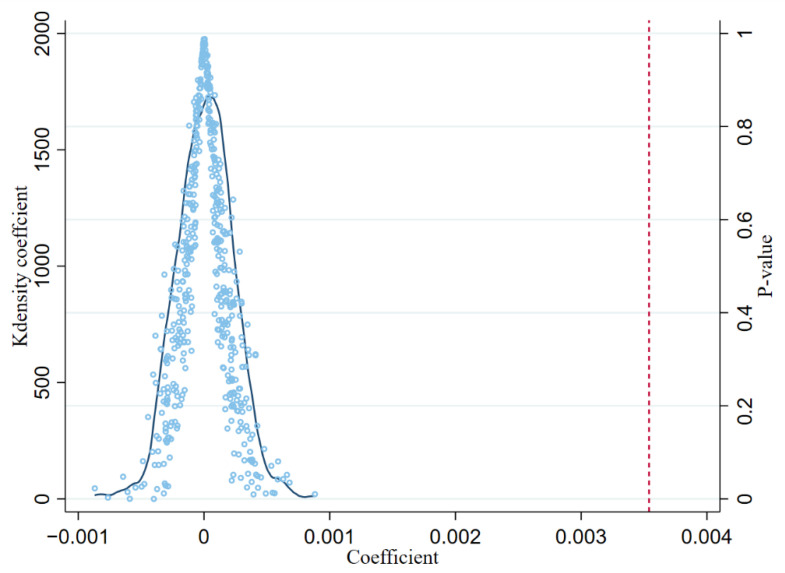
Placebo test.

In the baseline regression, although this paper controlled for a large number of individual characteristic variables with year fixed effects, the health deterioration effect due to the trade shock may still be affected by unobservable factors in the individual-year dimension, leading to biased estimation results. In this paper, a placebo test was conducted by randomly selecting the treatment group along with the year of the policy shock [46]. Specifically, using a computer program randomly assigned to each prefecture, a year within the sample period (excluding both ends) was randomly selected as the year of the spurious policy shock, and 500 times this operation was performed to observe the estimated coefficients and p-value distribution. If the policy is not affected by unobservable factors, the random treatment does not affect the corresponding value, and the mean treatment effect of the 500 estimated spurious policy shocks should converge with a probability of 0. The results of the placebo test are presented visually in Figure 2. As can be seen, the spurious policy shock effects in the stochastic process are all concentrated around zero, and most p-values are greater than 0.1. In addition, the baseline regression estimates in this paper under real policy shocks are far from the spurious policy effects and are significant outliers, which indicates that the empirical results in this paper are unlikely to be affected by omitted variable bias to a substantial degree.

### 5.3. Robustness Tests

#### 5.3.1. Indicator Change

Although interfered with by various non-measurable factors, the observational indicators can provide a more comprehensive response to the health status of micro-individuals. Therefore, this paper remeasures the health status of residents by using the results of health self-assessment in the CHNS questionnaire. The subjective health indicators are assigned a value of 0 if the respondents perceive their health to be “very good” and “good” compared to their peers, and 1 if it is “fair” and “poor”. The regression results are reported in column (1) of Table 4. The greater the tariff differential experienced by a region, the higher the probability that its self-assessed health results are fair and poor after China’s accession to the WTO, which further proves the reliability of the core findings of this paper. Consistent with Facchini, Liu, Mayda and Zhou [33], this paper uses the prefecture´s value of exports to the United States in 2001 to weight the independent variable. As shown in column (2) of Table 4, the estimation results are robust.

#### 5.3.2. Two-Period DID

In this section, the sample is divided into two time periods straddling the policy shock, 1993–2000 and 2004–2011. Within the two subperiods, all variables are averaged for the two-period DID test. This method can be used to mitigate potential serial correlation problems and thus avoid overstatement of the estimated coefficients and significance of the core explanatory variables [42]. Although this method reduces the degrees of freedom of the regression sample, it also improves the confidence of the DID causal identification. As shown in column (3) of Table 4, the estimate of the DID test variable remains significantly positive at the 1% level for both periods, and the estimates in the baseline regression of this paper remain robust.

#### 5.3.3. Controlling for Interferences from Other Sources

While China’s WTO accession caused a trade shock, the health status of the population may also be affected by a variety of other policies. First, the global financial crisis in 2008 may have seriously affected the normal life of the population and thus interfered with the estimation results of the health of the population in this paper, for which the sample interval is reduced to 1993–2006 and re-estimated. As the results in column (4) of Table 4 show, the regression coefficients are still significant. Second, the most likely policy that interferes with the results of this paper during the same period is the sharp reduction of foreign investment restrictions in China in 2002. Indeed, related studies also provide much empirical evidence on the impact of foreign investment entry on the health of the population. In this regard, as shown in column (5) of Table 4, this paper further introduces an interaction term between the dummy variable for provinces with high foreign capital entry and the dummy variable for the corresponding policy year in the model. Third, in 2006, the Chinese government issued a “five-year target” for energy conservation and emission reduction, which requires provinces to reduce chemical oxygen demand (COD) and sulfur dioxide emissions (SO_2_). To control for this policy shock, this paper follows the method of Fan, Lin and Lin [32] and adds to the model the interaction terms of the dummy variables for the year of each province’s pollution reduction target and policy. The results in column (6) of Table 4 show that after controlling for additional environmental policies, the DID model in this paper still captures the adverse effects of trade shock in each region on the health of the local population. In addition, column (7) also controls for the number of graduates (lngra) versus actual employment (lnemploy) in 2000 for each province, and the results remain robust.

#### 5.3.4. Prefecture-Specific Trends

Although this paper has controlled for many factors that may confound the estimation results, changes in individual health status may also be influenced by some unobservable prefecture-specific factors in their area, causing different trends in the outcome variables in the treatment and control groups. To avoid bias in the estimation results due to potential prefecture-specific trends, borrowing from Liu and Qiu [47], an interaction term of the prefectural category with the time trend characterizing the prefecture-specific variables is added to the model for estimation, and the regression results are shown in column (8) of Table 4. The core interaction term remains significantly positive after controlling for the prefecture-specific linear time trend, which shows that the unobservable prefecture-specific factors do not materially affect the core findings of this paper.

#### 5.3.5. Weighted Regression

The baseline regression data in this paper are derived from individual-level survey data distributed across 41 cities, and the sample selection bias arising from the unequal distribution of survey respondents across cities should be considered. Column (9) of Table 4 reports the results of the DID estimation using the population density of each prefecture as the weight in order to prevent large cities from dominating estimation results. Once again, the core findings of this paper are unaffected.

## 6. Heterogeneity Impact Analysis

### 6.1. Type of Residence

Up to this point, China’s accession to the WTO has been found to have a significant negative impact on its residents’ health status. However, due to great differences in factor endowments and development levels, the country´s urban and rural areas are a source of heterogeneity. Due to lack of regional data regarding the perceived change of uncertainty, the place of residence is used to distinguish urban from rural areas. Columns (1) and (2) of Table 5 show that trade shock hurts the health status of both urban and rural Chinese residents, but the effect is greater among urban residents and insignificant among rural residents. The possible explanation is that urban areas offer greater mobility and denser labor markets, both of which serve to increase employment opportunities. While residents of urban areas apparently were at the “epicenter” of the shock caused by trade liberalization, they were first to reap its dividends.

### 6.2. Gender

Are there gender differences in the adverse effects of trade shock on the health of the population? To explore this effect, the baseline model is estimated separately for female and male individuals. Columns (3) and (4) of Table 5 indeed point to a gender difference in that the coefficient pertaining to the change in uncertainty indicates a health loss in the male subsample only. Reasons for this phenomenon are: first, women are more inclined to consume more in terms of nutrition and health compared to men, thus weakening the adverse effects of trade liberalization on health. Second, unlike male workers who favor manual labor, female workers have a comparative advantage in labor involving cognitive skills, while intense physical labor is a crucial factor in the health impairment of men. Third, the existence of gender hiring bias in the labor market worsens the employment status of female labor and reduces the relative working hours of female workers, thus mitigating the health effects of the trade shock.

### 6.3. Geography

Considering the vast size of China, different geographic regions often have differences in location, infrastructure level, policy tilt, and culture, thus we explore the impact of geographic location on the core findings of this paper. The results in columns (5) and (6) of Table 5 show that the trade shock only has a significant negative impact on the health status of residents in coastal areas. This is because, after China’s accession to the WTO, a series of trade policy adjustments were made, including the reduction of tariff and non-tariff barriers, the granting of rights to engage in foreign trade, and the improvement of relevant laws and regulations in the field of foreign trade. The distinctive features of these policy adjustments favoring the eastern coastal areas has strengthened the impact of trade liberalization on these areas.

### 6.4. Testing for Nonlinearity

The OLS estimation performed up to this point excludes nonlinear variation in the treatment effects. To test for this possibility, the sample is divided in five categories defined by change in uncertainty, in analogy to Facchini, Liu, Mayda and Zhou [33]. This calls for the introduction of four interaction terms TScity×Post02×Group, with Group the pertinent categorical variable. The results are shown in Table 6. It is not difficult to find that the health deterioration effect of trade shock is mainly contributed by cities in the second, third, and fifth groups, a finding that also confirms the existence of nonlinear characteristics of its effect.

## 7. Mechanism

In this section, three channels of influence linking trade liberalization to individual health are distinguished, using the full period of observation 1991–2011.

### 7.1. Migration

For individuals who decide to migrate in response to trade liberalization, their hukou status is of great importance. If they lack an urban hukou, they do not have access to the city´s health insurance scheme. The CHNS survey registers the hukou as 1 if the resident has an urban hukou and 0 if they have a rural hukou. In columns (1) and (2) of Table 7, the sample is split into the categories hukou and nonhuku, with the former indicating the presence of urban hukou. We find that the health status of nonhukou group is more adversely affected by the trade shock. Considering the massive internal migration due to trade shock [33], the inequality of opportunity caused by not obtaining an urban hukou would further increase the probability of mental and physical illness among migrant workers.

### 7.2. Working Hours

Another channel of influence is (excessive) working hours. The CHNS survey also records respondents´ average weekly working hours, and the regression results are reported in column (3) of Table 7. This shows that hours indeed rose after China’s accession to the WTO, an increase also found among workers in Cambodia following an export shock [48]. Such a shock encourages an expansion of production; however, labor-saving technology typically cannot be adopted in the short term. In an attempt to alleviate the surge in the cost of labor, many firms take advantage of the increased local competition for jobs by demanding longer hours, in neglect of labor laws. However longer working hours reduce the time available for rest and exercise, thus causing adverse effects on workers´ health.

### 7.3. Environmental Pollution

A third channel of influence affecting residents´ health is environmental pollution, which is known to have increased in response to the export expansion following China´s accession to the WTO [4]. The Atmospheric Composition Analysis Group at Dalhousie University (Canada) provides satellite PM2.5 pollutant data that can be used to monitor the prefectures of China. This allows a matching of pollution information with the indicator of the change in uncertainty after 2001 derived in Section 4 above. The pertinent coefficient of 0.06459 (see column (4) of Table 7) supports the notion that pollution emissions increased after China´s accession to the WTO, a finding corroborated by Bombardini and Li [4]. The negative effects of increased environmental pollution in terms of health status, life expectancy, and mortality have been documented in [49,50].

## 8. Conclusions

While many studies have highlighted the export boom resulting from trade liberalization, the results of this paper suggest that China’s WTO accession has caused hidden health costs. China’s accession to the WTO and the permanent normal trade relations treatment granted by the U.S. constituted a trade shock for most Chinese residents that may have affected their labor market outcomes. This paper develops a measure of this change in tariff uncertainty at the prefecture level and links it to changes in health status at the individual level using the DID approach, with the following main findings: First, the trade shock caused by WTO accession significantly worsened the health status of the residents. Second, the trade shock affected the health status of residents by inducing rural non-household residents to migrate, extending residents’ working hours, and exacerbating regional pollution emissions. Third, urban residents, male residents, and coastal residents were more sensitive to trade shocks, and further analysis suggests that there may be a nonlinear relationship between regional trade shock and residents’ health status.

The findings of this paper have important implications for policy makers. Existing research has generally concluded that trade liberalization caused by China’s WTO accession can boost exports, product quality, total factor productivity, and innovation, while ignoring health costs of residents. Although China’s WTO accession will improve firm performance in many dimensions, it may also have negative effects on workers. Therefore, in implementing trade liberalization policies, policy makers should consider the possible health costs.

Due to data availability, there are two drawbacks in this study, and we will overcome them in future studies. First, CHNS only includes 41 cities in Shanghai, Beijing, Shandong, Guangxi Zhuang Autonomous Region, Jiangsu, Henan, Hubei, Hunan, Guizhou, Liaoning, Chongqing, and Heilongjiang Provinces. Second, this paper uses self-assessment of health and blood pressure measurements as proxy variables for the health of residents. Although subjective health self-assessment indicators can provide a more comprehensive measurement of individuals’ overall health, the existence of individual heterogeneity may affect the accuracy. Similarly, blood pressure indicators ensure accuracy and objectivity while sacrificing comprehensiveness.

## Figures and Tables

**Table 1:** Data and statistical description of variables.

Variable	Observations	Average	SD	Min	Max
health	61,106	0.136	0.343	0	1
TScity	61,106	5.002	8.509	0.148	51.506
gender	61,106	0.488	0.5	0	1
lnage	61,106	3.536	0.668	0	4.615
eduback	61,106	1.647	1.388	0	6
insurance	61,106	0.48	0.5	0	1
marry	61,106	1.949	0.705	1	5

**Table 2:** Baseline specification results.

	(1)	(2)	(3)
	*health*	*health*	*health*
TScity×Post02	0.00278 ***	0.00402 ***	0.00354 ***
	(0.00057)	(0.00067)	(0.00083)
gender		0.03810 ***	0.03814 ***
		(0.00555)	(0.00555)
lnage		0.17831 ***	0.17831 ***
		(0.00987)	(0.00989)
eduback		–0.02716 ***	−0.02717 ***
		(0.00264)	(0.00263)
insurance		0.02064 **	0.02058 **
		(0.00742)	(0.00734)
marry		0.00407	0.00412
		(0.00487)	(0.00485)
lnfinv00×Post02			0.00753
			(0.00526)
Prefecture FE	YES	YES	YES
Year FE	YES	YES	YES
N	61,106	55,320	55,320
Adjusted R^2^	0.024	0.107	0.107

Note: Clustering standard errors at the province level are in parentheses: ** and *** indicate significance at the 5% and 1% levels, respectively. The number of observations differs across columns due to the presence of some missing control variables.

**Table 3:** Dynamic and expectation effect.

	(1)	(2)	(3)
	Dynamic Effect	Expectation Effect	
TScity×Year1997	0.00091		
	(0.00156)		
TScity×Year2000	0.00070		
	(0.00109)		
TScity×Year2004	0.00193 **		
	(0.00084)		
TScity×Year2006	0.00431 ***		
	(0.00132)		
TScity×Year2009	0.00712 ***		
	(0.00156)		
TScity×Year2011	0.00342 **		
	(0.00155)		
TScity×Post02		0.00254 ***	0.00370 ***
		(0.00070)	(0.00091)
TScity×Pre1		−0.00056	0.00034
		(0.00092)	(0.00118)
Controls	YES	NO	YES
Prefecture FE	YES	YES	YES
Year FE	YES	YES	YES
N	51,098	61,106	55,320
Adjusted R^2^	0.107	0.024	0.107

Note: Clustering standard errors at the province level are in parentheses: ** and *** indicate significance at the 5% and 1% levels, respectively. The estimated models in columns (1) and (3) include control variables that measure the level of regional public service provision in addition to the full set of individual characteristic variables.

**Table 4:** Robustness tests.

	(1)	(2)	(3)	(4)	(5)	(6)	(7)	(8)	(9)
	Subjective Health	Weighted Health	Two-Period DID	Financial Crisis	FDI Liberalization	Environmental Policy	Employment	Prefecture-Specific Trends	Weighted Regression
TScity×Post02			0.00353 ***	0.00287 ***	0.00215 **	0.00310 ***	0.00264 **	0.00271 ***	0.00269 ***
			(0.00067)	(0.00075)	(0.00085)	(0.00095)	(0.00093)	(0.00079)	(0.00077)
TSexport×Post02	0.00103 ***	0.00211 **							
	(0.00017)	(0.00066)							
FDItreat×Post02					0.02317	0.02461 *		0.02464 *	0.02488 **
					(0.01336)	(0.01298)		(0.01292)	(0.01071)
COD×Post06						0.00001		0.00007	0.00042
						(0.00187)		(0.00184)	(0.00180)
${\mathrm{SO}}_{2}\times Post06$						−0.00204 **		−0.00209 ***	−0.00118
						(0.00067)		(0.00066)	(0.00078)
lngra00×Post02							−0.02487	−0.02353	0.00520
							(0.03111)	(0.03571)	(0.04334)
lnemploy00×Post02							−0.00001	−0.00001	0.00002
							(0.00010)	(0.00010)	(0.00011)
city×Year								−0.00001	−0.00004
								(0.00003)	(0.00003)
Controls	YES	YES	YES	YES	YES	YES	YES	YES	YES
Prefecture FE	YES	YES	YES	YES	YES	YES	YES	YES	YES
Year FE	YES	YES	YES	YES	YES	YES	YES	YES	YES
N	52,422	29,919	16,604	30,185	55,320	55,320	55,320	55,320	51,098
Adjusted R^2^	0.107	0.136	0.162	0.108	0.107	0.107	0.107	0.107	0.104

Note: Clustering standard errors at the province level are in parentheses: *, ** and *** indicate significance at the 10%, 5%, and 1% levels, respectively. FDItreat is a dummy variable that assigns a value of 1 to the prefecture located in a province if it is more open to foreign investment than the average value, and 0 otherwise.

**Table 5:** Heterogeneity test results.

	(1)	(2)	(3)	(4)	(5)	(6)
	Urban	Rural	Female	Male	Coastal Areas	Other Areas
TScity×Post02	0.00535 ***	0.00285	0.00132	0.00423 ***	0.00341 **	0.00358
	(0.00169)	(0.00267)	(0.00087)	(0.00100)	(0.00119)	(0.00296)
Controls	YES	YES	YES	YES	YES	YES
Prefecture FE	YES	YES	YES	YES	YES	YES
Year FE	YES	YES	YES	YES	YES	YES
N	20,592	34,728	28,842	26,478	25,889	29,431
Adjusted R^2^	0.114	0.105	0.121	0.097	0.108	0.108

Note: Clustering standard errors at the province level are in parentheses: ** and *** indicate significance at the 5% and 1% levels, respectively.

**Table 6:** Testing for nonlinearity.

Variables	*health*
Group1: TScity×Post02	0.05524
	(0.03661)
Group2: TScity×Post02	0.05086 **
	(0.02192)
Group3: TScity×Post02	0.03318 **
	(0.01294)
Group4: TScity×Post02	0.01126
	(0.00827)
Group5: TScity×Post02	0.00552 ***
	(0.00141)
Controls	YES
Prefecture FE	YES
Year FE	YES
N	55,320
Adjusted R^2^	0.107

Note: Clustering standard errors at the province level are in parentheses: ** and *** indicate significance at the 5% and 1% levels, respectively.

**Table 7:** Testing for channels of influence on health status.

	(1)	(2)	(3)	(4)
	*health*		*hours*	*pollutant*
	*non*-*hukou*	*hukou*		
TScity×Post02	0.00523 **	0.00281 *	0.57991 **	0.06459 **
	(0.00177)	(0.00145)	(0.24857)	(0.02802)
lngra00×Post02	−0.20980 ***	0.09785 **	3.75144	2.92683 ***
	(0.03371)	(0.03597)	(5.22026)	(0.38732)
lnfinv00×Post02	0.00487	−0.17561 *	−6.57101	−2.78619 ***
	(0.01953)	(0.07166)	(5.28266)	(0.44092)
lnemploy00×Post02	0.00044 ***	0.00027 **	0.00854	0.01412 ***
	(0.00002)	(0.00009)	(0.00605)	(0.00123)
city×Year	−0.00007	0.00001	−0.00299	0.00017
	(0.00014)	(0.00005)	(0.00550)	(0.00012)
Controls	YES	YES	YES	YES
Prefecture FE	YES	YES	YES	YES
Year FE	YES	YES	YES	YES
N	24,052	28,987	26,247	5472
Adjusted R^2^	0.040	0.044	0.095	0.939

Note: Clustering standard errors at the province level are in parentheses: *, ** and *** indicate significance at the 10%, 5%, and 1% levels, respectively. Employment opportunities are not only directly related to health, but are also strongly associated with the three mechanisms proposed in this paper. To control for employment effects, we include the logarithm of the number of college graduates and workers in each region in the regression. The effects of these variables in log form are calculated in the same way as in Section 4.2 for the variable of age.

## Data Availability

CHNS analyzed in this study is a publicly available dataset, which can be found here: https://www.cpc.unc.edu/projects/china/. Satellite PM2.5 pollutant data is also publicly available and can be found here: https://sites.wustl.edu/acag/datasets/surface-pm2-5/#V5.GL.02. China Regional Statistical Yearbook and China Industrial Enterprises Database are available with permission of the National Bureau of Statistics of China. China Customs Database is available with permission of the General Administration of Customs.

## References

[1] Deaton A., Paxson C. (2001). Mortality, education, income, and inequality among American cohorts. Themes in the Economics of Aging.

[2] Rodriguez-Martinez A., Zhou B., Sophiea M.K., Bentham J., Paciorek C.J., Iurilli M.L., Carrillo-Larco R.M., Bennett J.E., Di Cesare M., Taddei C. (2020). Height and body-mass index trajectories of school-aged children and adolescents from 1985 to 2019 in 200 countries and territories: A pooled analysis of 2181 population-based studies with 65 million participants. Lancet.

[3] Levine D.I., Rothman D. (2006). Does trade affect child health?. J. Health Econ..

[4] Bombardini M., Li B. (2020). Trade, pollution and mortality in China. J. Int. Econ..

[5] Stevens P., Urbach J., Wills G. (2013). Healthy trade: The relationship between open trade and health. Foreign Trade Rev..

[6] Handley K. (2014). Exporting under trade policy uncertainty: Theory and evidence. J. Int. Econ..

[7] Handley K., Limao N. (2015). Trade and investment under policy uncertainty: Theory and firm evidence. Am. Econ. J. Econ. Policy.

[8] Handley K., Limao N. (2017). Policy uncertainty, trade, and welfare: Theory and evidence for China and the United States. Am. Econ. Rev..

[9] Feng L., Li Z., Swenson D.L. (2017). Trade policy uncertainty and exports: Evidence from China’s WTO accession. J. Int. Econ..

[10] Hu G., Liu S. (2021). Economic Policy Uncertainty (EPU) and China’s export fluctuation in the post-pandemic era: An empirical analysis based on the TVP-SV-VAR model. Front. Public Health.

[11] Imbruno M. (2019). Importing under trade policy uncertainty: Evidence from China. J. Comp. Econ..

[12] Zhao T. (2022). Economic policy uncertainty and manufacturing value-added exports. Eng. Econ..

[13] Pierce J.R., Schott P.K. (2016). The surprisingly swift decline of US manufacturing employment. Am. Econ. Rev..

[14] Gilchrist S., Sim J.W., Zakrajšek E. (2014). Uncertainty, Financial Frictions, and Investment Dynamics.

[15] Ouyang D., Yuan W. (2022). Industrial Development and Trade Policy Uncertainty: Evidence from China’s WTO Accession. https://ssrn.com/abstract=4188516.

[16] Carballo J., Handley K., Limão N. (2018). Economic and Policy Uncertainty: Export Dynamics and the Value of Agreements.

[17] Yu M. (2015). Processing trade, tariff reductions and firm productivity: Evidence from Chinese firms. Econ. J..

[18] Amiti M., Konings J. (2007). Trade liberalization, intermediate inputs, and productivity: Evidence from Indonesia. Am. Econ. Rev..

[19] Liu Q., Ma H. (2020). Trade policy uncertainty and innovation: Firm level evidence from China’s WTO accession. J. Int. Econ..

[20] Schott P., Pierce J., Schaur G., Heise S. (2017). Trade policy uncertainty and the structure of supply chains. Proceedings of the 2017 Meeting Papers.

[21] Shepotylo O., Stuckatz J. (2017). Quantitative Text Analysis of Policy Uncertainty: FDI and Trade of Ukrainian Manufacturing Firms.

[22] Greenland A., Ion M., Lopresti J. (2019). Exports, investment and policy uncertainty. Can. J. Econ./Rev. Can. D’économique.

[23] Bartik T.J. (1991). Who Benefits From State and Local Economic Development Policies.

[24] Owen A.L., Wu S. (2007). Is trade good for your health?. Rev. Int. Econ..

[25] Herzer D. (2017). The long-run relationship between trade and population health: Evidence from five decades. World Econ..

[26] Adda J., Fawaz Y. (2020). The health toll of import competition. Econ. J..

[27] McManus T.C., Schaur G. (2016). The effects of import competition on worker health. J. Int. Econ..

[28] Guerrico S.F. (2021). The effects of trade-induced worker displacement on health and mortality in Mexico. J. Health Econ..

[29] Tanaka M. (2020). Exporting sweatshops? Evidence from Myanmar. Rev. Econ. Stat..

[30] Lang M., McManus T.C., Schaur G. (2019). The effects of import competition on health in the local economy. Health Econ..

[31] Dix-Carneiro R., Kovak B.K. (2017). Trade liberalization and regional dynamics. Am. Econ. Rev..

[32] Fan H., Lin F., Lin S. (2020). The hidden cost of trade liberalization: Input tariff shocks and worker health in China. J. Int. Econ..

[33] Facchini G., Liu M.Y., Mayda A.M., Zhou M. (2019). China’s “Great Migration”: The impact of the reduction in trade policy uncertainty. J. Int. Econ..

[34] Wang F., Milner C., Scheffel J. (2021). Labour market reform and firm-level employment adjustment: Evidence from the hukou reform in China. J. Dev. Econ..

[35] Johansson M., Partanen T. (2002). Role of trade unions in workplace health promotion. Int. J. Health Serv..

[36] Gardner J., Oswald A.J. (2007). Money and mental wellbeing: A longitudinal study of medium-sized lottery wins. J. Health Econ..

[37] Sullivan D., Von Wachter T. (2009). Job displacement and mortality: An analysis using administrative data. Q. J. Econ..

[38] Burgoon B., Raess D. (2009). Globalization and working time: Working hours and flexibility in Germany. Politics Soc..

[39] Steinmetz H., Schmidt P. (2010). Subjective health and its relationship with working time variables and job stressors: Sequence or general factor model?. Work Stress.

[40] Lovely M., Popp D. (2008). Trade, Technology, and the Environment: Why Have Poor Countries Regulated Sooner?.

[41] Managi S., Hibiki A., Tsurumi T. (2009). Does trade openness improve environmental quality?. J. Environ. Econ. Manag..

[42] Bertrand M., Duflo E., Mullainathan S. (2004). How much should we trust differences-in-differences estimates?. Q. J. Econ..

[43] Brandt L., Van Biesebroeck J., Wang L., Zhang Y. (2017). WTO accession and performance of Chinese manufacturing firms. Am. Econ. Rev..

[44] Kemptner D., Jürges H., Reinhold S. (2011). Changes in compulsory schooling and the causal effect of education on health: Evidence from Germany. J. Health Econ..

[45] Angrist J.D., Pischke J.-S. (2008). Mostly Harmless Econometrics.

[46] La Ferrara E., Chong A., Duryea S. (2012). Soap operas and fertility: Evidence from Brazil. Am. Econ. J. Appl. Econ..

[47] Liu Q., Qiu L.D. (2016). Intermediate input imports and innovations: Evidence from Chinese firms’ patent filings. J. Int. Econ..

[48] Robertson R. (2000). Trade liberalisation and wage inequality: Lessons from the Mexican experience. World Econ..

[49] Chen Y., Ebenstein A., Greenstone M., Li H. (2013). Evidence on the impact of sustained exposure to air pollution on life expectancy from China’s Huai River policy. Proc. Natl. Acad. Sci. USA.

[50] Tanaka S. (2015). Environmental regulations on air pollution in China and their impact on infant mortality. J. Health Econ..

